# ALA Pretreatment Improves Waterlogging Tolerance of Fig Plants

**DOI:** 10.1371/journal.pone.0147202

**Published:** 2016-01-20

**Authors:** Yuyan An, Lin Qi, Liangju Wang

**Affiliations:** College of Horticulture, Nanjing Agricultural University, Nanjing 210095, Jiangsu, China; Estación Experimental del Zaidín (CSIC), SPAIN

## Abstract

5-aminolevulinic acid (ALA), a natural and environmentally friendly plant growth regulator, can improve plant tolerance to various environmental stresses. However, whether ALA can improve plant waterlogging tolerance is unknown. Here, we investigated the effects of ALA pretreatment on the waterlogging-induced damage of fig (*Ficus carica* Linn.) plants, which often suffer from waterlogging stress. ALA pretreatment significantly alleviated stress-induced morphological damage, increased leaf relative water content (RWC), and reduced leaf superoxide anion (${\text{O}}_{2}{}^{\bar{\cdot }}$) production rate and malonaldehyde (MDA) content in fig leaves, indicating ALA mitigates waterlogging stress of fig plants. We further demonstrated that ALA pretreatment largely promoted leaf chlorophyll content, photosynthetic electron transfer ability, and photosynthetic performance index, indicating ALA significantly improves plant photosynthetic efficiency under waterlogging stress. Moreover, ALA pretreatment significantly increased activities of leaf superoxide dismutase (SOD) and peroxidase (POD), root vigor, and activities of root alcohol dehydrogenase (ADH), and lactate dehydrogenase (LDH), indicating ALA also significantly improves antioxidant ability and root function of fig plants under waterlogging stress. Taken together, ALA pretreatment improves waterlogging tolerance of fig plants significantly, and the promoted root respiration, leaf photosynthesis, and antioxidant ability may contribute greatly to this improvement. Our data firstly shows that ALA can improve plant waterlogging tolerance.

## Introduction

Under natural conditions, plants are frequently exposed to transient or long-term soil waterlogging, which has long been identified as a major abiotic stress [1,2]. Soil waterlogging significantly limits growth, development, and survival of numerous plant species, not only in natural ecosystems but also in agricultural and horticultural systems [3,4]. Exploring effective approaches is of urgent need to improve plant waterlogging tolerance.

Application of bio-regulators (plant growth regulators as well as endogenous plant hormones) to plants is one effective way to enhance plant stress tolerance [5]. 5-Aminolevulinic acid (ALA) is a key precursor of all porphyrin compounds such as chlorophyll and heme in plants [6]. In 1998, hormonal activities of ALA were found in plant tissue culture [7]. Recently, more research indicates ALA is not only an important intermediate in biological metabolism, but also a vital plant growth regulator [6]. ALA regulates several key physiological processes, including promoting seed germination [8], improving photosynthesis [9], contributing to plastid-to-nucleus signaling [10], and enhancing stress tolerance [6]. These results suggest great application potential of ALA in plant production. One of ALA’s outstanding physiological roles is increasing plant stress tolerance. For example, exogenous ALA significantly increased cold resistance in rice (*Oryza sativa*) [11], melon (*Cucumis melo*) [9], soybean (*Glycine max*) [12], and pepper (*Capsicum annuum*) [13] seedlings. Likewise, ALA could remarkably enhance salt tolerance of cotton [14], pakchoi (*Brassica campestris*) [8], potato (*Solanum tuberosum*) [15], date palm (*Phoenix dactylifera*) [16], oilseed rape (*Brassica napus*) [17], and cucumber (*Cucumis sativus*) seedlings [18]. ALA is also effective in improving plant tolerance to low light [9], drought [19], and heat stresses [20]. Thus, ALA can promote plant tolerance to multiple abiotic stresses. As mentioned above, waterlogging is also a serious abiotic stress. However, little research has been devoted to the effect of ALA on plant waterlogging tolerance.

Stressful conditions often limit plant growth. It has been well-documented that ALA improves plant growth, not only under normal conditions [21], but more notably upon environmental stresses [9, 11, 19, 20, 22]. Improvement of photosynthesis contributes mainly to growth promotion by ALA [9, 11, 19, 20, 22]. However, no information is available on whether ALA can improve plant photosynthetic capacity under waterlogging stress. Chlorophyll fluorescence, a probe of plant photosynthesis in vivo, has been widely used for many years to monitor the photosynthetic performance of plants [23]. Therefore, in this study, chlorophyll fluorescence kinetics were employed to evaluate the effect of ALA on photosynthesis and its possible mechanisms under waterlogging.

Almost all types of abiotic stress, including waterlogging, are accompanied by an increased production of reactive oxygen species (ROS) such as superoxide radical (${\text{O}}_{2}{}^{\bar{\cdot }}$). Since ROS are highly reactive and toxic, overproduction of ROS will damage plant cells irreversibly by oxidation of cellular components [24]. Malondialdehyde (MDA) is the final product of membrane lipid peroxidation. Therefore, MDA content is often used to assess the extent of oxidative damage of cell plasma membrane [25]. To scavenge damaging ROS, plants have evolved antioxidant enzymes such as superoxide dismutase (SOD), peroxidase (POD), catalase (CAT), and non-enzymatic antioxidants like ascorbate and glutathione [24]. Enhancement of antioxidant capacity and reduction of oxidative damage have been reported as a critical mechanism behind ALA-induced resistance to multiple stresses such as salt [26], drought [19], and heat [20]. Therefore, we hypothesized that ALA might mitigate the damaging effect of waterlogging by stimulating the antioxidant defense system as well.

Waterlogging blocks the oxygen supply to the roots, which inhibits respiration and hence greatly reduces the energy status of cells [1]. Therefore, one of the best characterized responses for plants under waterlogging is the metabolic switch from oxidative phosphorylation to anaerobic fermentation in roots to maintain ATP prodution [27,28]. The fermentation pathways are not used under aerobic conditions, but quickly activated by low oxygen conditions, suggesting a positive role in waterlogging adaptation mechanism. Kennedy et al. [29] showed that plants which had more active fermentation pathways were more waterlogging tolerant. Ethanol and lactic acid are two main fermentation pathways in plants during waterlogging, where alcohol dehydrogenase (ADH) and lactate dehydrogenase (LDH) are two key enzymes, respectively [1]. Yang et al. [30] have reported that ALA mitigates salinity stress-induced suppression of plant respiratory activity and improves salt tolerance. Whether ALA can improve anaerobic fermentation pathways and consequently contribute to maintaining metabolic activities in roots under waterlogging is not known.

*Ficus carica* (fig, Moraceae) is an important crop because of its nutrient rich fruits [31] as well as its medicinal [32,33] and ornamental value [34]. Fig trees often suffer from waterlogging, because they are mainly grown in tropical and subtropical countries where a higher probability of long-term waterlogging and flash floods occur commonly because of extreme rainfall events [35]. In this study, we first determined the mitigating effect of exogenous ALA on waterlogging- induced damage to fig plants by analyzing leaf relative water content (RWC), superoxide radical (${\text{O}}_{2}{}^{\bar{\cdot }}$) production rate, and malonaldehyde (MDA) level. Then, we explored the mechanisms how ALA enhances fig waterlogging tolerance by investigating the antioxidant defense system in the leaves, root vigor, and fermentative enzyme activities in the roots. Based on the results obtained, we discussed the mechanism underlying improvement of waterlogging tolerance by ALA.

## Materials and Methods

### Plant materials and stress imposition

In early April, one-year-old fig *(Ficus carica* Linn. cv. *Masui Dauphine)* plants, with mean height and diameter of 35.0 cm and 1.3 cm, respectively, were transplanted into plastic pots (30 cm diameter and 25 cm height) filled with a mixed cultural substrate that contained ≥50% organic matter, ≥2.5% nitrogen, ≥2.5% phosphorus, and ≥2.5% potassium. There were 30 pots in total and two plants in each pot. Plants were watered every two days to make the soil water content 70%-80% of field capacity before the beginning of treatments. On July 15, plants were randomly separated into five groups for different treatments: (1) control, (2) waterlogging, (3) 5 mg/L ALA pretreatment + waterlogging (ALA1), (4) 10 mg/L ALA pretreatment + waterlogging (ALA2), and (5) 20 mg/L ALA pretreatment + waterlogging (ALA3). There were 6 pots for each treatment. Water content of the control group was kept at 70%-80% of field capacity during the whole experiment period. All waterlogging pots, with or without ALA pretreatment, were irrigated twice per day so that the water in each pot kept at a level of 3 cm above the substrate surface. ALA pretreatment was carried out by root-irrigating each pot with 500 mL of ALA solution on June 25, 20 days before the initiation of waterlogging, when the same amount of water was irrigated to each pot in the other two treatments. Plants were cultured under a rain shelter with natural condition in the campus of Nanjing Agricultural University (32°02'N, 118°50'E). The atmospheric temperature during the experiment was 28–34°C during the day and 20–26°C at night. Photosynthetically active radiation reached a daytime peak of 1,200 μmol**·**m^-2^·s^-1^. Chlorophyll fast fluorescence characteristics were monitored in vivo and physiological indexes were determined using the middle leaves on the 0, 2^nd^, 4^th^, and 6^th^ d of waterlogging. Root vigor and root enzyme activities were measured using fine roots on the 4^th^ and 6^th^ d of waterlogging.

### Measurement of RWC and chlorophyll contents in fig leaves

Relative water content (RWC) was determined as (FW-DW)/(TW-DW)×100, where FW is the fresh weight, DW is the dry weight after oven drying at 80°C for 24 h, and TW is the turgid weight after re-hydration for 6 h in complete darkness at 4°C. Leaf chlorophyll (a and b) were extracted by 95% ethanol and determined according to Lichtenthaler and Wellburn [36].

### Measurement of antioxidant enzyme activities and oxidative damage

Random 0.1 g fresh leaves were homogenized with 2 mL 50 mM phosphate buffer (pH 7.8) in a pre-chilled mortar and pestle on ice. The homogenate was centrifuged at 12,000 × *g* at 4°C for 20 min. The supernatant was collected and used for determination of SOD (EC 1.15.1.1) and POD (EC 1.11.1.7) activity

SOD activity was assayed by the photochemical NBT method [37]. Three milliliter of assay mixture contained 50 mM phosphate buffer (pH 7.8), 130 mM methionine, 750 mM NBT, 100 mM EDTA, 100 mL of enzyme extract, and 20 mM riboflavin. The reduction of NBT was monitored at 560 nm. One unit of SOD activity was defined as the quantity of SOD required to inhibit the photo-reduction of NBT by 50%. POD activity was assayed strictly according to the methods described by An and Liang [37]. One unit of POD activity was defined as the amount of enzyme that made OD_470_ increase 0.01 per min.

Superoxide radical production rate was measured by monitoring the nitrite formation from hydroxylamine in the presence of ${\text{O}}_{2}{}^{\bar{\cdot }}$. Fresh leaf samples were homogenized with 4 mL of 65 mM potassium phosphate buffer (pH 7.8) and centrifuged at 10,000 *g* for 10 min. The incubation mixture contained 0.9 mL of 65 mM phosphate buffer (pH 7.8), 0.1 mL of 10 mM hydroxylamine hydrochloride and 1 mL of the supernatant. After incubation at 25°C for 20 min, 17 mM of sulfanilamide and 7 mM of 1-naphthylamine were added to the incubation mixture. Then, trichloromethane in the same volume was added and centrifuged at 5,000 *g* for 5 min. The OD_530_ of the aqueous solution was read using a spectrophotometer. A standard curve with NO_2_^-^ was used to calculate the production rate of ${\text{O}}_{2}{}^{\bar{\cdot }}$ from the chemical reaction of ${\text{O}}_{2}{}^{\bar{\cdot }}$ and hydroxylamine.

The MDA content was expressed as mmol per gram of fresh weight. Random 0.2 g fresh leaf tissue was homogenized and then added to 2.0 mL of 5% trichloroacetic acid (TCA) and centrifuged at 10,000 × *g* for 10 min. 2.0 mL of supernatant was added to 2.0 mL of 0.67% 2-thiobarbituric acid (TBA). The mixture was heated at 100°C for 30 min and then quickly cooled in an ice bath. After centrifugation at 10,000 × *g* for 10 min to remove suspended turbidity, the absorbance of the supernatant was measured at 532 nm. The value for non-specific absorption at 600 nm was subtracted.

### Measurement of proline content

Proline content was determined following the method described by Bates [38] with slight modification. Fresh leaf tissue (0.5 g) was placed in the test tube and added to 5 mL of 3% sulfosalicylic acid. After covering the glass stopper, the mixture was heated at 100°C for 10 min and then cooled to room temperature. Two milliliter of supernatant was transferred to another test tube with glass stopper, and 2 mL of glacial acetic acid and 3 mL of acidic ninhydrin was added. The mixture was further heated at 100°C for 40 min and quickly cooled. The reaction solution was then added to 5 mL of toluene and shooked fully. After static hierarchy, the supernatant was centrifuged at 3,000 × *g* for 5 min. Then the absorbance of the supernatant was measured at 520 nm.

### Chlorophyll a fast fluorescence and OJIP test

Chlorophyll fast fluorescence transient was measured by a Plant Efficiency Analyzer (Hansatech, UK) according to methods of Srivastava et al. [39]. All the leaves were immediately exposed to a saturating light pulse (3,000 μmol·m^-2^·s^-1^ PFD) for 1 s after dark adapted for 20 min. Each transient obtained from the dark-adapted samples was analyzed according to the OJIP-test [39].

### Measurement of root vigor

Fine roots (0.5 g) were placed in the petri dish and added to 5 mL of 0.4% TTC (2, 3, 5-Triphenyltetrazoliumchloride) and 5 mL 67 mM phosphate buffer (pH 7.0). The roots were completely immersed in the solution, and then heated it at 37°C for 1 h in the dark. After ending the reaction by adding 2 mL of 1 M H_2_SO_4_ to the solution, the tip section of roots were transferred to the test tube with stopper. 20 mL of methanol were added to let the roots completely immersed in the solution. The test tube were then incubated at 35°C until the tip section of roots become white and the extracting solution was measured at 486 nm.

### Measurement of ADH and LDH activity

The fine root tissues were harvested on the 4^th^ and 6^th^ day of waterlogging and frozen in liquid nitrogen, stored at -70°C until used. The root sample was extracted in 50 mM Tris-HCl (pH 6.8) containing 5 mM MgCl_2_, 5 mM mercaptoethanol, 15% (v/v) glycerol, 1 mM EDTA, 1 mM EGTA, and 0.1 mM Pefabloc proteinase inhibitor. The homogenate was centrifuged at 10,000 × *g* at 4°C for 20 min. The supernatant was collected and used for measurement of ADH (EC 1.1.1.1) and LDH (EC 1.1.1.27) activity. ADH was measured as described by Waters et al. [40] with a slight modification. The ADH assay reaction mixture contained 50 mM TES (pH 7.5), 0.17 M NADH, and started with 40% (v/v) acetaldehyde. LDH activity was determined specifically according to Mustroph and Albrecht [41].

### Statistical analysis

The pot cultures were carried out in completely randomized design. Statistical analysis was carried out with the SPSS statistical computer package (version 16.0, SPSS Inc., Chicago. IL). Data were compared statistically among different treatments by analysis of variance (ANOVA) followed by least significant difference tests (LSD) at the 0.05 and 0.01 level of confidence. Correlation analysis was carried out between leaf proline concentration and RWC or ${\text{O}}_{2}{}^{\bar{\cdot }}$ production.

## Results

### ALA alleviated morphological damage and leaf RWC reduction induced by waterlogging

**Fig 1:**
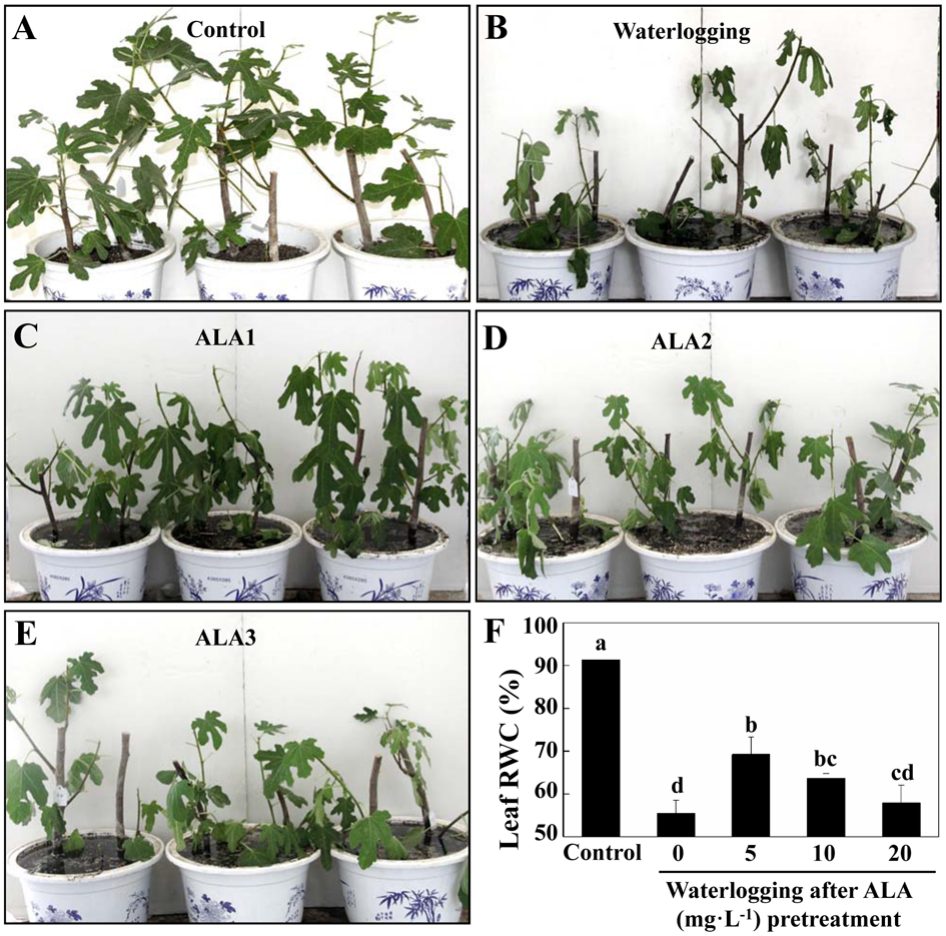
Effect of ALA pre-treatment on leaf morphology and RWC in fig plants under waterlogging. A to E: Leaf morphology on the 6^th^ day under different treatments. ALA1: Waterlogging after 5 mg·L^1^ ALA pretreatment. ALA2: Waterlogging after 10 mg·L^-1^ ALA pretreatment. ALA3: Waterlogging after 20 mg·L^-1^ ALA pretreatment. F: Leaf relative water content (RWC) on the 6^th^ day under different treatments. Data indicate mean ± standard deviation (SD) of six replicates (*n* = 6). The same letters at the top of each bar indicate no significant differences at *P*<0.05.

To estimate the effects of waterlogging and ALA on fig plants, leaf morphological characteristics and leaf RWC were monitored during the whole experiment. Fig plants wilted and leaves showed chlorosis, curling, and even abscising under waterlogging conditions for 6 days (Fig 1A and 1B). However, ALA-pretreatment mitigated the symptoms induced by waterlogging (Fig 1C and 1E), and the effect was the most effective under 5 mg**·**L^-1^ ALA pretreatment (Fig 1C). Waterlogging for 6 days significantly decreased leaf RWC in figs (Fig 1F, *P*<0.05). ALA-pretreatment partially inhibited the decline in RWC under waterlogging (*P*<0.05), and the effect of 5 mg**·**L^-1^ ALA again was the best, which increased leaf RWC by 24.82%, compared to waterlogging treatment. These results indicated ALA pretreatment alleviated the damaging effects of waterlogging stress on fig plants.

### ALA reduced leaf ${\text{O}}_{2}{}^{\bar{\cdot }}$ production, MDA, and proline content under waterlogging

**Fig 2:**
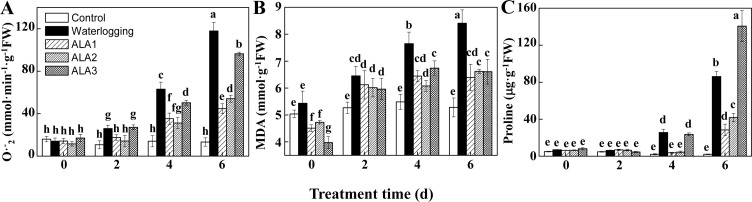
ALA pretreatment reduces leaf ${\text{O}}_{2}{}^{\bar{\cdot }}$ production rate, MDA, and proline content under waterlogging. A to C: Dynamic changes of leaf superoxide anion production rate (${\text{O}}_{2}{}^{\bar{\cdot }}$, A), malondialdehyde (MDA, B), and proline content (C) under different treatments. ALA1: Waterlogging after 5 mg·L^1^ ALA pretreatment. ALA2: Waterlogging after 10 mg·L^-1^ ALA pretreatment. ALA3: Waterlogging after 20 mg·L^-1^ ALA pretreatment. Data indicate mean ± standard deviation (SD) of six replicates (*n* = 6). The same letters at the top of each bar indicate no significant differences at *P*<0.05.

To confirm the mitigating effect of ALA on waterlogging stressed fig plants, we determined the dynamic change of leaf ${\text{O}}_{2}\bar{\cdot }$ production and MDA content. Compared with the controls, leaf ${\text{O}}_{2}\bar{\cdot }$ production rate increased significantly under waterlogging condition (Fig 2A), up to 8.90-fold greater than the control on the 6^th^ day of waterlogging. ALA pretreatment, however, slowed the increasing rate of ${\text{O}}_{2}\bar{\cdot }$ production. On the 6^th^ day, leaf ${\text{O}}_{2}\bar{\cdot }$ production in plants pretreated with 5 mg**·**L^-1^, 10 mg**·**L^-1^, and 20 mg**·**L^-1^ ALA were 62.07%, 54.08%, and 18.19%, respectively, lower than that in waterlogged plants without ALA pretreatment (*P*<0.05). During the whole experimental period, ${\text{O}}_{2}\bar{\cdot }$ production rate in 10 mg**·**L^-1^ ALA-pretreated plants was similar to that in 5 mg**·**L^-1^ ALA-pretreated ones, but the latter was significantly lower than the former on the 6^th^ day (*P*<0.05). These results indicated that waterlogging-induced increase of leaf ${\text{O}}_{2}\bar{\cdot }$ production was suppressed by ALA pretreatment, and 5 mg**·**L^-1^ ALA was the most effective.

Similar to the ${\text{O}}_{2}\bar{\cdot }$ production rate, MDA content increased significantly after waterlogging treatment (Fig 2B, *P*<0.05), and ALA pretreatment reduced the leaf MDA content in waterlogged plants, which were significantly different on the 4^th^ and 6^th^ day (*P*<0.05). No significant differences were found among effects of ALA at 5 mg**·**L^-1^, 10 mg**·**L^-1^, and 20 mg**·**L^-1^ during the waterlogging period. These results indicated waterlogging-induced increase of MDA content could be suppressed by ALA pretreatment, and there was no significant dose-effect from 5 to 20 mg**·**L^-1^.

Proline is an amino acid accumulating in plants exposed to a wide variety of environmental stresses [42]. In our experiment, waterlogging significantly stimulated proline accumulation after the 4^th^ day (Fig 2C, *P*<0.05). However, proline accumulation was significantly negatively correlated to RWC (*r* = -0.990; *P* = 0.010), but positively correlated to ${\text{O}}_{2}\bar{\cdot }$ production rate (*r* = 0.972; *P* = 0.028). Therefore, proline accumulation in fig plants exposed to waterlogging was likely to be an indicator of stress- induced damaging effect. The pretreatment with 5 mg**·**L^-1^ and 10 mg**·**L^-1^ ALA, especially 5 mg**·**L^-1^, significantly reduced the proline accumulation on the 4^th^ and 6^th^ day of waterlogging (*P*<0.05), while 20 mg·L^-1^ ALA increased proline accumulation on the 6^th^ day (Fig 2C). These results suggested that proline accumulation in waterlogged fig plants was probably a passive stress indicator, and ALA pretreatment at low concentration could inhibit waterlogging-induced proline accumulation.

These results together indicate that ALA pretreatment mitigates the damaging effects of waterlogging, and 5 mg·L^-1^ is the most efficient concentration. Therefore, 5 mg·L^-1^ ALA was used for ALA pretreatment in the following experiments.

### ALA improved chlorophyll content in fig leaves under waterlogging

**Fig 3:**
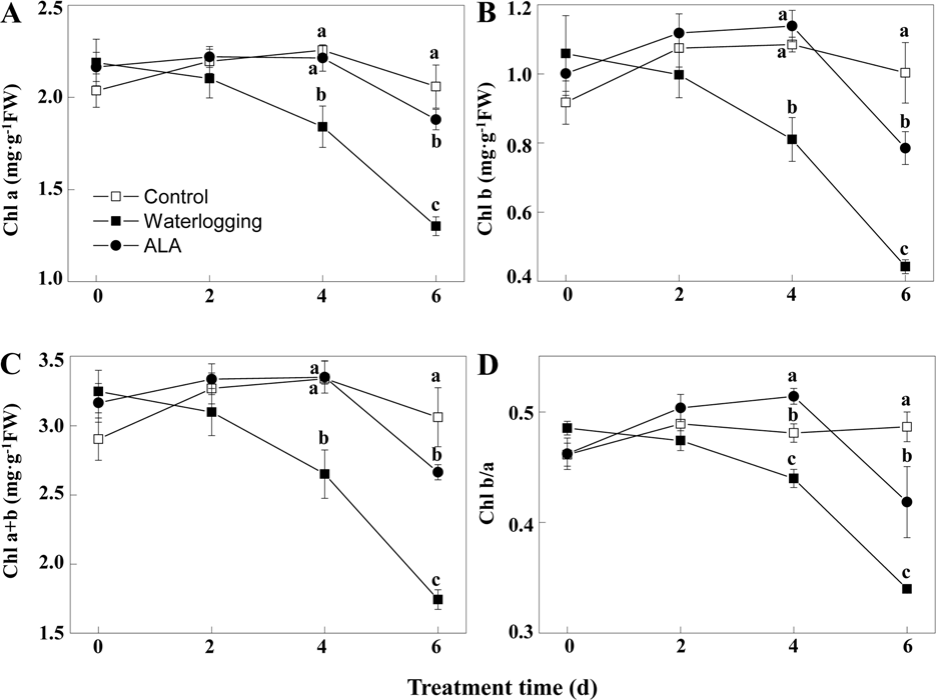
ALA pretreatment increases chlorophyll content and chlorophyll b/a under waterlogging. A to D: Dynamic changes of chlorophyll a (Chl a, A), chlorophyll b (Chl b, B), total chlorophyll (Chl a+b, C), and the ratio of chlorophyll b/a (Chl b/a, D) under different treatments. ALA: Waterlogging after 5 mg·L^-1^ ALA pretreatment. Data indicate mean ± standard deviation (SD) of six replicates (*n* = 6). Different letters on the same time point indicate significant differences at *P*<0.05.

To explore how ALA mitigates the damaging effects of waterlogging, we monitored the dynamic changes of chlorophyll, whose reduction could cause chlorosis. Waterlogging significantly reduced the content of Chl a, Chl b, Chl a+b, and ratio of Chl b/a after the 4^th^ day of treatment and these effects became more severe at the late stage of stress (Fig 3, *P*<0.05). Compared with waterlogging treatment alone, ALA pretreatment significantly increased Chl a, Chl b, Chl a+b content and Chl b/a ratio (Fig 3, *P*<0.05). At the end of the experiment, Chl a, Chl b, Chl a+b content, and Chl b/a ratio in fig leaves pretreated with ALA increased by 45%, 78%, 53%, and 23%, respectively, compared to waterlogging alone (*P*<0.05). These results demonstrated that ALA pretreatment improved leaf chlorophyll levels under waterlogging stress.

### ALA improved chlorophyll fast fluorescence characteristics under waterlogging

**Fig 4:**
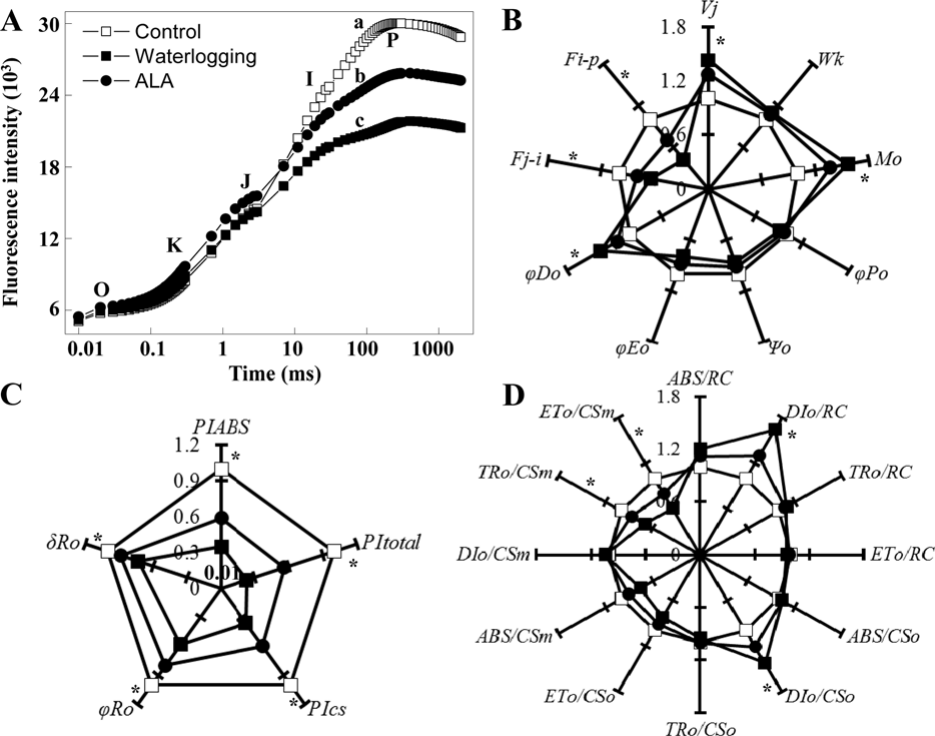
Effect of ALA pre-treatment on leaf chlorophyll fast fluorescence characteristics under waterlogging. A: Leaf chlorophyll a fast fluorescence transient on the 6^th^ day under different treatments. In the O-K-J-I-P nomenclature, O stands for the origin (minimum), K- I are intermediate inflections, and P is for peak. Different small letters indicate significant differences between I-P phase of different treatments at *P*<0.05. B to D: Leaf chlorophyll fluorescence parameters on the 6^th^ day under different treatments. ALA: Waterlogging after 5 mg·L^-1^ ALA pretreatment. *V*_j_: Relative variable fluorescence intensity at the J-step. *W*_k_: Amplitude of the K step as a parameter of the PSII donor side. *M*_o_: Approximated initial slope of the fluorescence transient. *φP*_o_: Maximum quantum yield for primary photochemistry. *ψ*_o_: Probability that a trapped exciton moves an electron into the electron transport chain beyond Q_A_^-^. *φE*_o_: Quantum yield for electron transport. *φD*_o_: Quantum yield for heat dissipation. *F*_i-p_: Slow PQ pool reduced ability. *F*_j-I_: Fast PQ pool reduced ability. *PI*_total_: Performance index for energy conservation from excitation to the reduction of PSI end acceptors. *PI*_ABS_: Performance index on absorption basis. *PI*cs: (Performance index on cross section basis. *δR*_o_: Probability with which an electron from the intersystem electron carriers moves to reduce end electron accepters at the PSI acceptor side. *φR*_o_: Quantum yield for reduction of end electron accepters at the PSI acceptor side. *ABS/RC*: Absorption flux per reaction center (RC). *DIo/RC*: Dissipated energy flux per RC. *TRo/RC*: Trapped energy flux per RC. *ETo/RC*: Electron transport flux per RC. *ABS/CSo*: Absorption flux per cross section (CS, at *t* = 0). *DIo/CSo*: Dissipated energy flux per CS (at *t* = 0). *TRo/CSo*: Trapped energy flux per CS (at *t* = 0). *ETo/CSo*: Electron transport flux per CS (at *t* = 0). When *t* = *t*_Fmax_, *CSo* was replaced by *CSm*. In figure B-D, * near each parameter indicate significant differences at *P*<0.05 between treatments. Data indicate mean ± standard deviation (SD) of 6 replicates (*n* = 6).

Chlorophyll fluorescence is widely used to evaluate effects of stresses on plant photosynthetic efficiency. To examine the effects of waterlogging and ALA pretreatment on photosynthetic efficiency of fig leaves, we investigated chlorophyll fast fluorescence characteristics in fig leaves on the 6^th^ day of treatment. Waterlogging significantly reduced the prompt fluorescence intensity between I and P phase which was, however, significantly recovered by ALA pretreatment (Fig 4A, *P*<0.05).

A multi-parametric radar plot with the quantification of some selected parameters (relative to the controls) allowed an accurate analysis of the stress situation (Fig 4B and 4D). *M*_o_ represents the maximum rate of Q_A_ reduction. *φD*o is quantum yield of heat dissipation, and *V*j is relative variable fluorescence at J phase. These three parameters in fig leaves increased significantly under waterlogging (Fig 4B, *P*<0.05). Compared with the waterlogging treatment alone, ALA-pretreatment significantly reduced the increases of these three parameters (*P*<0.05), suggesting ALA decreased Q_A_ reduction rate and non-photochemical energy dissipation, and increased the open state in the PSII reaction center in fig leaves under waterlogging stress. Waterlogging significantly decreased *F*_i-p_ and *F*_j-I_ which, however, were largely recovered by ALA-pretreatment (Fig 4B, *P*<0.05). These results indicated ALA- pretreatment improved the reducing ability of the fast and slow PQ pool in fig leaves under waterlogging stress. *PI*_total_, *PI*_ABS_, and *PI*cs, three photosynthetic performance indexes, significantly decreased on the 6^th^ day of waterlogging (*P*<0.05), whereas ALA-pretreatment significantly alleviated the decreases induced by waterlogging (Fig 4C, *P*<0.05). These results indicated ALA-pretreatment improved the photosynthetic performance in both PSI and PSII reaction centers of fig leaves under waterlogging stress. Furthermore, waterlogging reduced *δR*_o_ in fig leaves, but this process was also partially recovered by ALA-pretreatment (Fig 4C, *P*<0.05). *DI*_*o*_*/RC* and *DI*_o_*/CS* reflect the amount of dissipated energy. *DI*_*o*_*/RC* and *DI*_o_*/CS* increased under waterlogging condition (Fig 4D, *P*<0.05), whereas ALA-pretreatment significantly inhibited waterlogging effects on these two parameters (*P*<0.05). ALA pretreatment also significantly inhibited the decline of *ETo/CSm* and *TRo/CSm* under waterlogging, indicating ALA improved photosynthetic electron transport activity and ability of trapping energy in fig plants under waterlogging.

### ALA enhanced SOD and POD activities in fig leaves under waterlogging

**Fig 5:**
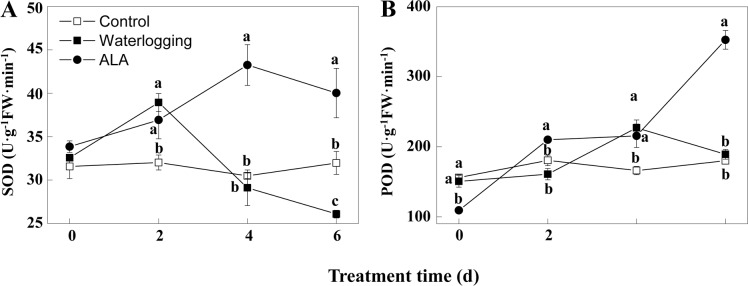
ALA pretreatment increases leaf SOD and POD activities under waterlogging. A, B: Dynamic changes of superoxide dismutase (SOD, A) and perioxidase (POD, B) under different treatments. ALA: Waterlogging after 5 mg·L^-1^ ALA pretreatment. Data indicate mean ± standard deviation (SD) of six replicates (*n* = 6). Different letters on the same time point indicate significant differences at *P*<0.05.

Compared with the control, SOD activity in leaves increased significantly on the 2^nd^ day and then decreased sharply with time under waterlogging stress (Fig 5A). However, SOD activity in ALA-pretreated waterlogged plants kept higher levels than that in the control plants during the whole experimental period. No significant differences were found in SOD activities of waterlogged plants with and without ALA pretreatment on the 2^nd^ day, but the latter was significantly higher than the former on the 4^th^ and 6^th^ day (*P*<0.05).

Leaf POD activity increased significantly on the 4^th^ day of waterlogging compared to the control (*P*<0.05), and no significant differences were found on the other time points (Fig 5B). Compared with the waterlogging treatment alone, ALA pretreatment significantly increased leaf POD activity on the 2^nd^ and 6^th^ day of waterlogging (*P*<0.05). These results together indicated ALA pretreatment enhances SOD and POD activities under waterlogging stress.

### ALA improved root vigor and root respiratory metabolism under waterlogging

To further investigate the effect of ALA pretreatment on flooding tolerance of fig plants, root vigor and fermentative enzyme activities including ADH and LDH were determined.

**Fig 6:**
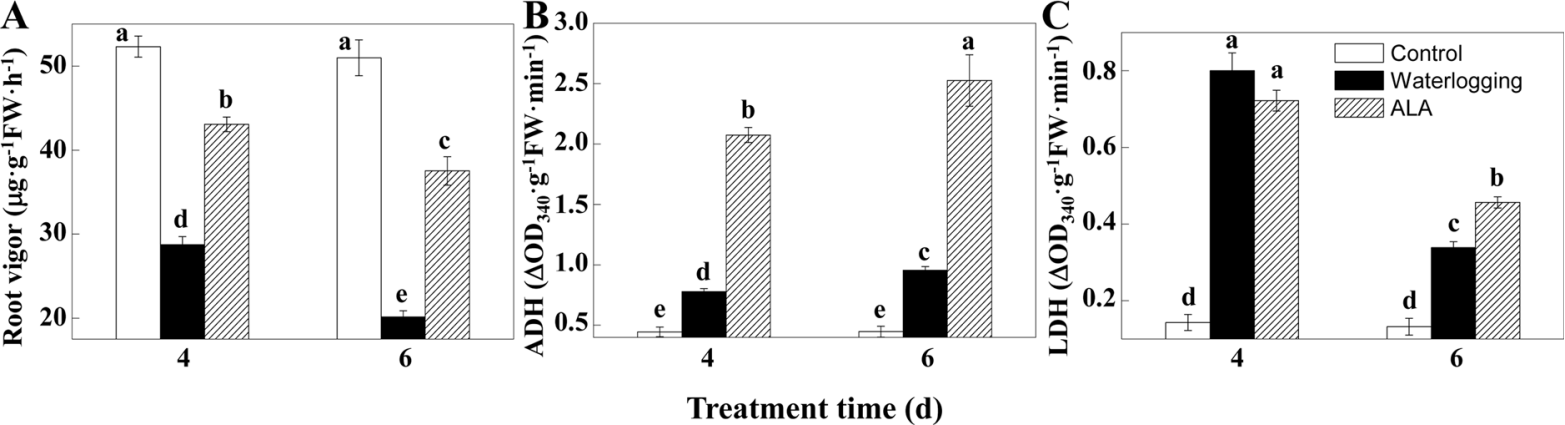
Effect of ALA pre-treatment on root vigor and root fermentative enzyme activities in fig plants under waterlogging. A: Root vigor on the 4^th^ and 6^th^ day. B: Alcohol dehydrogenase (ADH) activities on the 4^th^ and 6^th^ day. C: Lactate dehydrogenase (LDH) activities on the 4^th^ and 6^th^ day. Data indicate mean ± standard deviation (SD) of six replicates (*n* = 6). Different letters on the same time point indicate significant differences at *P*<0.05.

Compared to the control, root vigor of fig plants was significantly reduced by 45.05% and 60.50%, respectively, on the 4^th^ and 6^th^ day of waterlogging (Fig 6A, *P*<0.05). ALA pretreatment significantly increased root vigor in waterlogged fig plants (*P*<0.05). Root vigor in ALA pretreated plants were 50% and 86%, respectively, higher than that in the waterlogged plants on the 4^th^ and 6^th^ day. These results suggested ALA pretreatment improved root vigor of fig plants under waterlogging stress.

ADH activity increased notably under waterlogging stress on the 4^th^ and 6^th^ day (*P*<0.05), which was further significantly promoted by ALA pretreatment (Fig 6B, *P*<0.05). The ADH activity in ALA pretreated seedlings were 2.6 and 5.7 times higher than that in the waterlogged and control plants, respectively, on the 6^th^ day of experiment.

Compared to the control, LDH activity under waterlogging condition increased on the 4^th^ day (5.59-fold of controls, *P*<0.05) and then decreased (by 57.66%) on the 6^th^ day (Fig 6C, *P*<0.05). Compared to the waterlogging treatment alone, ALA pretreatment slightly reduced LDH activity on the 4^th^ day, but significantly increased it on the 6^th^ day (*P*<0.05), indicating ALA pretreatment partially promoted LDH activity under waterlogging stress. The promoting effect of ALA on ADH activity was much greater than that on LDH activity.

## Discussion

Fig trees, important as fruit, medicinal, and ornamental plants [32,33], are widely spread in tropical and subtropical countries. As it is native in dry and sunny regions, even in rocky areas [33,34], fig trees are sensitive to waterlogging stress. Waterlogging has become one of the most critical limits for fig growth and production [35]. In addition, more frequent waterlogging conditions are expected worldwide due to more heavy precipitation [43,44]. Therefore, it is important for fig trees to improve waterlogging tolerance. It has been well established that ALA, a natural plant growth regulator, could effectively improve plant tolerance to many environmental stresses, including cold, heat, salt, drought, and low light [6]. In the present study, we found ALA pretreatment significantly increased leaf RWC (Fig 1) and decreased leaf ${\text{O}}_{2}\bar{\cdot }$ production and MDA content (Fig 2), suggesting ALA mitigates the damaging effect of waterlogging on fig trees. Indeed, much less waterlogging stress symptoms were found from ALA-pretreated fig plants under waterlogging conditions (Fig 1). These results indicate ALA pretreatment is an effective method for improving waterlogging tolerance of fig trees. ALA is a natural amino acid present in all living cells, and it is biodegradable and harmless for crops, humans, and animals [45]. Therefore, improving waterlogging tolerance of fig trees with ALA is likely to meet modern environmental and food quality guidelines, suggesting its great application potential. According to our results, among the three tested concentrations, 5 mg·L^-1^ ALA could be the optimal concentration for fig plants.

Leaf RWC is widely used to indicate plant water balance [25], since it expresses the relative amount of water in plant tissues. Waterlogging stress often resulted in reductions in leaf RWC of plants [46]. Similarly, leaf RWC in fig trees significantly decreased under waterlogging stress. However, ALA pretreatment significantly increased leaf RWC in waterlogged fig plants, indicating ALA helps maintain water balance in fig plants. This can be reflected by less wilting of ALA- pretreated fig plants (Fig 1). Root water uptake is one of the major components of plant water balance [2]. In the present study, ALA pretreatment significantly improved root vigor of fig plants under waterlogging stress, indicating ALA may stabilize fig roots and enhance their water uptake capacity. This may be an important mechanism for ALA-pretreated fig plants to partially maintain water balance under waterlogging stress.

Osmotic adjustment also contributes to maintaining water balance of plants [47]. Proline is traditionally recognized as a compatible solute [48]. However, whether proline accumulation has any adaptive value or is merely a passive stress indicator has been controversial [48–50]. The actual reason behind proline accumulation under stressed conditions seems to be dependent on plant species and stress types. Here, proline accumulation was significantly negatively correlated to RWC in leaves under waterlogging stress, indicating proline did not contribute to maintaining water balance. The significantly positive correlation between proline concentration and ${\text{O}}_{2}\bar{\cdot }$ production suggests proline accumulation may be an indicator of injury in fig leaves under waterlogging stress. Proline concentration under waterlogging with ALA pretreatment, which is the lowest under 5 mg·L^-1^ ALA and highest under 20 mg·L^-1^ ALA, further indicates proline accumulation is probably a reflection of stress-induced injury. The exact role of proline accumulation in waterlogged fig plants, and the mechanism behind ALA- induced decline of proline accumulation under waterlogging stress need to be further studied.

Waterlogging inhibits root mitochondrial respiration due to oxygen deficiency [44,51]. Plants under waterlogging shift their metabolism from oxidative phosphorylation to anaerobic fermentation to maintain ATP production [28,52]. This shift is followed by fermentation of pyruvate to the major end products, ethanol or lactate, yielding NAD^+^ to sustain anaerobic metabolism [53]. Lactate is toxic for the cells and leads to acidification of the cytosol, however, lactate production in plants is a transient process, and a quantitative correlation does not exist between lactate production and cytoplasmic acidification [54]. Therefore, lactate production may be less harmful to viability than supposed. Ethanol production may also be disadvantageous, but interest in the possibility of ethanol as a cause of cell death during anoxia has diminished with the recognition that ethanol levels usually found in plants are not enough to cause toxicity [55]. Moreover, ethanol can be almost totally diffused out of the tissues to the surrounding solution where it is diluted or metabolized by microorganisms [56]. The switch in fermentation pathway, from lactate to ethanolic fermentation, has been recognized as an evolved mechanism for surviving hypoxia without extensive cell damage [55,57]. There is growing evidence of the important roles of lactate and ethanolic fermentation in improving waterlogging tolerance by using loss-of-function mutants and over-expressed lines for genes encoding ADH, PDC, and LDH [29,54,57,58,59,60]. Here, both ADH and LDH activities were significantly promoted by ALA pretreatment under waterlogging stress, suggesting that anaerobic fermentation is activated by ALA to supply energy for plant growth and viability. Improvement of ADH activity by ALA pretreatment was more significant than LDH activity, indicating ALA pretreatment also contributes to change from lactate fermentation to alcoholic fermentation, which represents an important indicator of plant ability to survive hypoxia without extensive cell damage [55]. Significant enhancement in root vigor by ALA pretreatment indicates ALA improves root anaerobic fermentation activity without resulting in root injury. Taken together, promotion of fermentation pathways, especially ethanol pathway, is an important mechanism for improvement of waterlogging tolerance by ALA.

Chlorophyll loss, a common consequence of waterlogging stress, was largely prevented in fig leaves by ALA. Since ALA is a key precursor of chlorophyll [6], application of exogenous ALA provides more precursors for chlorophyll biosynthesis. In addition, oxygen radicals have been considered as a major reason for chlorophyll destruction by waterlogging [61]. ALA significantly reduced the production of ${\text{O}}_{2}\bar{\cdot }$ and MDA accumulation in leaves of waterlogged fig plants. Therefore, ALA also prevents the loss of chlorophyll by decreasing the production of oxygen radicals. One potential consequence of chlorophyll destruction is the decrease of photosynthesis. Waterlogging stress often restricts plant photosynthesis [51]. It has been well documented that ALA improves plant photosynthesis under various stress conditions, including cold [11], salt [22], low light [9], water deficit [19], and heat [20]. In the present study, ALA significantly improved the electron transfer ability and photosynthetic performance index in waterlogged fig plants, indicating ALA can also improve plant photosynthesis under waterlogging stress. The maintenance of chlorophyll and photosynthesis is another mechanism for improvement of waterlogging tolerance by ALA.

Like many other types of stresses, waterlogging will accelerate the production of ROS and invoke oxidative stress in plants [51,62]. Here, ALA significantly decreased ${\text{O}}_{2}\bar{\cdot }$ production rate and MDA content, and largely enhanced activities of antioxidant enzymes, indicating ALA pretreatment promotes antioxidant capacity and reduces lipid peroxidation damage in waterlogged fig plants. Not only under waterlogging condition, many researches have reported that ALA can also improve plant antioxidant capacity and reduce oxidative damages under many other stress conditions, such as water deficit [19], heat [20], and salt [26]. Therefore, promoting antioxidant defense ability may be a common mechanism for ALA to improve plant tolerance to various abiotic stresses.

In summary, the data presented here show that ALA pretreatment mitigates the damaging effects of waterlogging and improves waterlogging tolerance of fig plants. The maintenance of root vigor, root respiration, and leaf photosynthesis, and the promotion of antioxidant ability play important roles in ALA-conferred waterlogging tolerance. Since ALA is a natural, nontoxic, biodegradable, and environmentally friendly plant growth regulator, and it improves plant photosynthesis and various abiotic stress tolerance widely, application of ALA to fig plants is expected to contribute greatly to the promotion of plant production.
